# Forensic imaging: a powerful tool in modern forensic investigation

**DOI:** 10.1080/20961790.2021.2008705

**Published:** 2022-03-07

**Authors:** Min Zhang

**Affiliations:** Faculty of Forensic Investigation Department of Criminal Justice, Coppin State University, Baltimore, MD, USA

**Keywords:** Forensic sciences, forensic imaging, forensic investigation, computed tomography (CT), magnetic resonance imaging (MRI), angiography, review

## Abstract

Forensic imaging is a non-invasive examination process during the forensic investigation. It is mainly used in forensic pathology as an adjunct to the traditional autopsy. In the past two decades, forensic imaging has been vigorously developed by forensic experts from computed tomography (CT) to multiple augmented techniques through CT and magnetic resonance imaging (MRI). The application field of forensic imaging has also been broadened as its advantages are recognised by more forensic practitioners. In addition to the forensic pathology, this technique has been used in other forensic disciplines, including forensic anthropology, forensic odontology, forensic ballistics and wildlife forensics, etc. This article reviews the development of forensic imaging as the practice and research development in different forensic disciplines based on the relevant literature analysis.

## Introduction

Modern forensic investigation utilises novel tools and advanced technologies to solve criminal and civil cases. Forensic imaging is obviously a powerful tool in this new era. Thanks to X-ray, computed tomography (CT), magnetic resonance imaging (MRI) and other medical imaging technologies, which have laid a solid foundation for the development of forensic imaging. Forensic imaging is the use of images to explain and document findings for forensic and medico-legal purposes [1]. It includes the X-ray, the multi-slice CT, MRI, the augmented minimally invasive techniques through CT and MRI such as angiography and biopsy, and three-dimensional (3D) surface scanning as an adjunct or alternative to the traditional invasive autopsy [1,2]. Because of its non-invasive features, postmortem imaging is often used in pathology instead of a traditional autopsy when the case is of sensitive religious concern, or a traditional autopsy is rejected by family members for other reasons [2]. In death investigations, postmortem imaging is also frequently applied prior to a traditional autopsy, in order to accurately locate traumas and pathological changes in the deceased [3]. In some traumatic deaths, such as fatal motor vehicle accidents [4], postmortem imaging has the ability to detect or presume fatal traumas. In the circumstances such as fatal traumas, an autopsy is not necessary if the forensic pathologists can determine the cause of death according to imaging results.

Since forensic imaging has been used in forensic investigation, it mainly focused on research and applications of forensic pathology [5,6]. It is worth noting that in recent years, this technique has been adopted in other forensic investigative disciplines including forensic anthropology [7], odontology [8], forensic ballistics [9], wildlife forensics [10] and clinical forensic medicine [2]. Clearly, forensic imaging has become a powerful tool for modern forensic investigation. This article summarizes the application and research progress on forensic imaging in different forensic investigative disciplines.

## The global impact of forensic imaging

In recent years, with the increasing recognition of its strengths, forensic imaging has been explored globally in forensic practice and research. For example, the forensic imaging research teams at the University Centre of Legal Medicine Lausanne-Geneva [1,11], the University of Berne [5] and the University of Zurich [8] in Switzerland, employed forensic imaging techniques, particularly CT and MRI in their outstanding research; the Chief Medical Examiner’s Office of Maryland in the United States utilises CT as an auxiliary method in autopsy [12]; the Armed Forces Institute of Pathology (disestablished in 2011, continues as American Institute of Radiologic Pathology) performed CT especially in gunshot and drown cases [13,14]; the Victorian Institute of Forensic Medicine in Australia [15] and the Institute of Forensic Medicine at University of Southern Denmark [16] performed CT scan along with standard autopsy in forensic cases; forensic pathologists in Italy made use of CT scan as a screening diagnostic test before conducting a traditional autopsy [17]; in Austria, researchers utilised forensigraphy imaging technique to detect relevant traces on and within the body of an examined person [18]; in Japan, 26 of 47 prefectures have at least one autopsy imaging centre with scanners that are dedicated for postmortem imaging [19]; the Academy of Forensic Science in China started conducting postmortem multi-slice computed tomography (PMCT) research since 2005 and has completed more than 500 forensic imaging cases with different causes of death [20,21], this team also contributed thin layer CT scanning and imaging reconstruction to estimate the age of teenagers through the sternal end of clavicle epiphyseal growth [22].

## The application of forensic imaging in different forensic disciplines

### Pre - CT time

Before the CT technology was invented in 1972 [23], the use of medical imaging in forensic investigation was not uncommon. X-ray was the primary imaging technique employed in forensics. As early as in the end of 19th century, X-ray was used for postmortem purposes [1]. In 1940s, stereoscopic radiography was utilised in hypertensive and ischemic heart disease deaths investigation in order to obtain pathological information [24]. Later, X-ray was widely used in forensic investigation, such as to assist in investigating the skull fracture mechanism [25] and the cause of death [26], to identify individuals [27,28] and study Egyptian mummy [29] in forensic anthropology, to develop the procedures and techniques in dental identification [30], to calculate gunshot size and pattern in forensic ballistics [31], to study the putrefaction which could cause radiographic postmortem changes in feline [32] and canine cadavers [33] in wildlife forensics, to investigate the injuries in industrial accidents, suicidal attempts and criminal assault of forensic living cases [34], etc.

Since CT and MRI were invented and used in the medical field in the 1970s, they have been increasingly used in forensic investigations.

### Forensic pathology

In 1979, Flodmark et al. [35] performed CT 10 days before autopsy in 90 neonates that had suffered perinatal hypoxia. They confirmed that findings at autopsy were correlated with the CT diagnosis. In 1987, Varnell et al. [36] found that CT could reveal the marked diffuse cerebral swelling with associated loss of gray-white differentiation in cyanide poisoning death, whereas this type of death may masquerade as natural disease and can be difficult to diagnose both clinically and pathologically. In 1999, Oliver et al. [37] demonstrated the advantages and pitfalls of CT in forensic pathological diagnosis of arterial gas embolism in fatal diving accidents, they pointed out that an understanding of pitfalls will aid in an accurate imaging diagnosis in diving accident cases.

Based on the development of CT and MRI in medicine, forensic imaging ushered in its improvement heyday. CT was proved to be an excellent visualisation tool with great potential for forensic documentation and evaluation of decomposed bodies [38]; it assisted in finding intrahepatic gas in deceased with the exceptions of putrefied or burned corpses [39]; it also showed the reliability in estimation of abdominal blood volume [40]. CT and MRI techniques were used to assist in correct examination of charred bodies [41]; CT and MRI showed comparable potential as forensic diagnostic tools for traumatic extra-axial hemorrhage, and revealed that MRI is more sensitive than CT for detection of subarachnoid hemorrhagic findings [42]; CT and MRI also assessed postmortem weights of liver and spleen accurately, and CT even could overcome the limitation of putrefaction and venous air embolism by the possibility to exclude gas, and in congestion cases, the imaging might be even more accurate than autopsy in weighing the livers [43].

After 2006, angiography was incorporated in forensic imaging. It was first tested in animal models for rapid vascular phenotyping [44,45]. Subsequently, a minimally invasive two-step postmortem angiographic technique was established with the first step of a bolus injection of oily contrast agent into human cadavers and the second step of radiographic imaging. Postmortem angiography enabled detailed examination of the vascular system which is difficult to be examined by traditional autopsy methods [46]. The wholebody postmortem MR angiography (PMMRA) was proved to be feasible [47]. However, pitfalls of postmortem CT angiography (PMCTA) were reported as a hemorrhagic pericardial effusion happened during the venous phase of angiography, and suggested it is necessary to critically analyse CT and PMCTA images in order to distinguish between artifacts, true pathologies and iatrogenic findings [48]. With this lesson in mind, in the subsequent studies, angiography incorporated forensic imaging emerged remarkable role in differentiation of hemopericardium due to ruptured myocardial infarction or aortic dissection on unenhanced CT [49]. In addition, researchers successfully applied angiography without traditional autopsy in a fatal bronchovascular fistula after lobectomy case [50] which further confirmed angiography is technically feasible in forensic pathology.

With the advent of the new approach which has the capability to perform 3D surface scans as well as postmortem image-guided bones and soft tissues biopsy, forensic imaging has developed into a 3D and micromorphology era. In 2010, Virtobot, a multi-functional robotic system for 3D scanning and automatic postmortem biopsy was first introduced to the research field [51]. In 2014, the second prototype of Virtobot updated the previous prototype, and the updated Virtobot is more accurate in biopsy and focuses on streamlining the workflow and increasing the level of automation [52]. Recently, a more advanced 3D scanning technology multispectral full-body imaging employed multispectral photogrammetry between 365 and 960 nm by utilising modified digital cameras, ultraviolet, near-infrared light sources and lens filters to visualise the latent evidence on the body such as latent bodily fluids and latent bruises [53].

### Forensic anthropology

CT imaging technology was widely used in forensic anthropology since 1980s. In 1981, Wong [54] was able to identify Hirschsprung disease in desiccated human remains by using CT. In 1993, Haglund and Fligner [55] successfully utilised CT to confirm human identification by comparing antemortem and postmortem skull CT scan scout views. In 1996, for facial reconstruction purposes, Phillips and Smuts [56] found the variation of the facial tissue thickness for the mixed racial population group of South Africa by CT scanning. In 1997, Quatrehomme et al. [57] proposed a new computerized methodology with the advantages of CT scan for facial reconstruction. By performing this new method, this research team was able to reconstruct by computer 3D facial model of the deceased. In 2006, Turner et al. [58] reported the first mathematical representation of the face continuum associated with given skull and collected a comprehensive CT head-scan database for forensic facial reconstruction, in order to assist in the identification of missing person and victims of violent crime. In the same period, Sidler et al. [59] proved that CT may be used as a valuable tool in disaster victim identification after a mass fatality incident because of the high efficiency of the technology.

By the end of the 20th century, forensic imaging application in forensic anthropology was elevated to a new level. In 2009, Harth et al. [60] applied Flat-Panel-CT eXplore Locus Ultra (eLU) system in determining the correlation between age and the stages of skull suture closure. The research team appraised that this method is useful in conjunction with other methods in age estimation. In 2014, López-Alcaraz et al. [61] applied CT on the pubic symphysis surface and the pubic body to relate them with age, and suggested that the image analysis of pubic bone offers a valid and alternative method for age estimation. In 2017, Ikeda [62] used Bayesian statistics in combination with CT imaging and suggested that they together can be used to estimate age at death based on costal cartilage calcification. Recently, Fan et al. [63] developed the CT image reconstruction of laryngeal cartilage and hyoid bone in adult age estimation using data mining methods. They suggested that this reconstruction should not be used alone in practice but can be used in combination with other indicators.

### Forensic odontology

Forensic odontology is the study of deceased’s dental records in order to identify the unknown individual. Dental radiology plays a critical role in forensic odontology. After forensic imaging was applied in forensic studies, researchers started utilising this technology in forensic odontology. In 2005, Jackowski et al. [64] introduced the application of CT in a burned corpse dental identification. They believe that transportable dental CT scanner can greatly help identify disaster victims and offers new possibilities of comparison of antemortem and postmortem dental information. In 2008, Jackowski et al. [65] successfully performed CT and 3D volume rendering to distinguish between dental ceramics and composite fillings and proved that this method is suitable for human dentition visualisation for forensic purposes. Bassed and Hill [66] utilised postmortem CT to determine the deceased children’s age by dentition in 2009 Victorian Bushfire disaster in Australia. They concluded that CT imaging is a useful tool for age estimation in certain conditions including the dental development can be obviously visualised and the presence or absence of restorations is irrelevant. In 2013, Franco et al. [67] observed the 3D reconstructions and CT slices of 103 postmortem full body CT and obtained optimal dental chart, which can serve as a valuable additional tool in the human dental identification. In 2014, Trochesset et al. [68] proposed the application of cone beam CT (CBCT) to forensic odontology, because the CBCT data sets can be displayed in three dimensions to visualise the dentition. They successfully generated intra-oral-like images from CBCT volumes and proved that these images are similar enough to traditional dental radiographs to allow for forensic dental identification. In 2015, Sakuma et al. [69] compared five corresponding anatomical reference points between postmortem CT images and dental original radiographs by superimposing the two types of images, and they found out there were significant anatomical differences in these two type images, which suggested odontology forensic imaging can aid in avoiding incorrect personal identification owing to erroneous information.

### Forensic ballistics

In forensic ballistics, forensic imaging is primarily used in the study of gunshot residues, shooting distance, foreign body’s location, and paths in the livings and deceased. In 2000, Stein et al. [70] conducted the gunshot wound forensic assessment research with CT application. In this research, caliber .38 Special, 357 magnum and 22 LR bullets were shot at experimental fresh pink skins from a range of 0 cm to 100 cm. The results suggested that CT records can differentiate between a contact shot and firing ranges of more than 10 cm. In 2003, Thali et al. [9] performed forensic imaging on eight gunshot fatalities, and the findings were confirmed by traditional autopsy that PMCT, MRI, 2D multi-planar reformation (MPR) and 3D shaded surface display (SSD) reconstruction have the capability to visualise the ballistic fracture pattern, the bullet track and localisation, trauma, pathological changes and gunshot residue deposition in a non-destructive method. In 2009, Puentes et al. [71] applied 3D-multislice computed tomography (MSCT) in a non-fatal gunshot case. In this case, the bullet’s trajectory was accurately determined by 3D-MSCT and this technology was proved to be helpful in estimating the victim and suspects locations in a multiple aggressor situation in crime scene investigation. In 2014, Maiese et al. [72] reported that PMCT and the 3D rendering of CT slice stack images not only helped with the wound path visualisation and bullet localisation, but also offered data for the crime scene reconstruction. In 2019, Gascho et al. [73] assessed the synergy of CT and MRI in gunshot wound cases with foreign bodies in head. They suggested that MRI provides a valuable supplement to postmortem CT for the detection of wound channel and soft tissue injuries. In 2020, Gascho et al. [74] emphasized the importance of MRI in gunshot case investigation. In one of the shooting investigations, MRI clearly showed the soft tissue injuries and the ruptured medulla oblongata, providing the investigators the graphic information on the death. Gascho et al. [75] also pointed out that special MRI sequences at 7 tesla MRI can delineate micro injury in soft tissue which could be easily ignored by macroscopical autopsy.

### Wildlife forensics

Although forensic imaging is not as widely used in wildlife forensics as human forensics, it has been reported in the field of wildlife. In 2008, Cooper and Cooper [76] proposed that imaging method can be used in forensic animal investigation. In 2008 and 2009, Heng et al. [32,33] applied X-ray on 41 feline animals and canine cadavers, and concluded that putrefaction could cause radiographic postmortem changes in animals. In 2015, Ribas [77] performed X-ray before forensic necropsy on a young female white-eared opossum (*Didelphis albiventris*). The images clearly revealed diffuse increased radio-opacity in both hemi-thoraces, and *in situ* necropsy findings confirmed bloody effusion in thorax cavity. In 2015, Frankenberg et al. [10] performed CT, PMCTA and MRI to determine the cause and manner of death in a 4-month-old deceased male fox. The results proved that the effects of semi-jacketed hunting ammunition in the fox were clearly revealed by forensic imaging without the need for any manual dissection. In 2018, Pankowski et al. [78] utilised X-ray and CT in a fatal common buzzard gunshot case. By performing imaging on the deceased buzzard, they estimated that the shot was taken from a smoothbore hunting gun with a probable 12 mm, 16 mm or 20 mm caliber and the buzzard died most likely as a result of spinal cord injury from a single shot of a hunting gun. In 2020, Hamel et al. [79] performed CT, MRI, necropsy, histology, culture, and molecular diagnostics on a freshly dead juvenile bottlenose dolphin, and these investigation methods together demonstrated disseminated coinfection of dolphin morbillivirus and *Aspergillus fumigatus*.

## Advantages, disadvantages and prospects

### Advantages

The advantages of forensic imaging are obvious. First, forensic imaging is the most suitable alternative of a traditional autopsy, especially when the case is religious sensitive, or the family member of the deceased cannot accept a traditional autopsy [2]. Second, high-resolution CT scan can be used to obtain the best quality images in forensic imaging because the radiation exposure issue can be neglected for postmortem examination [80]. Third, when performing the autopsy on the deceased who had an infectious disease such as tuberculosis [81] and coronavirus disease [82]. In this circumstance, forensic imaging can prevent practitioners from being directly exposed to uncertain pathogens, this irreplaceable advantage is particularly important during the global COVID-19 pandemic, suggesting the inestimable social value of the forensic imaging—the potential of being used in any unpredictable pandemic in the future. Fourth, in traditional experience-dependent investigations, forensic imaging can provide objective data to enhance the credibility of results. For example, in forensic anthropology and odontology age estimation, imaging data are a reliable reference in age determination by skeleton [60,61] and dentition [66]; in forensic odontology, imaging can be used to compare the dental repair material of the suspect with the suspicious material found at crime scene to support the evidence chain [65]. Fifth, true-to-scale 3D non-destructive gathering of forensic imaging findings can reveal the original injuries and pathological changes instead of destructing original evidence by surgical procedure. For example, in forensic ballistics, it is important to find out the bullet path in victims, and forensic imaging is ­definitely a fantastic method to investigate the original trajectory instead of destructively dissect the paths [71–74].

### Disadvantages

In order to apply forensic imaging objectively and correctly, the disadvantages of this technique should not be ignored. Forensic imaging has formally entered the field of forensics for nearly two decades, but due to its high cost, it is still not widely used in the daily work of forensics. The high costs include the instruments, the facility, the professional operator’s training, the hardware and software accessories and the maintenance [21,83]. In forensic pathology, sometimes imaging is unable to detect the pathological conditions in the deceased. For example, solely CT scanning has the limitation in detecting soft-tissue injury, MRI or 3D surface scanning are needed to assist in such cases [2]; forensic imaging did not reveal all the hemorrhagic sites in the brain injury, and a direct comparison between neuroimaging and forensic-neuropathological findings was necessary in this circumstance [42]; although forensic angiography enables detailed examination of the vascular system, PMCTA has the potential to induce the rupture during its performance [48], therefore attentions are needed in such cases. In forensic ballistic, forensic imaging shows its limitation in reacting to the metal foreign objects. For example, due to the metal artifacts caused by residual bullets, the radiological assessment of gunshot wounds on CT may be severely impeded [74]. In forensic anthropology and odontology, imaging is often used to assist in age estimation or human identification. However, the validity of forensic imaging is not confirmed yet, and it must be used as an aid together with other methods to estimate the age [60] or identify the unknown [67].

### Prospects

Due to the high cost of forensic imaging, it is not realistic for all forensic institutes to own the facility. In order to make better use of resources, a forensic imaging resource sharing platform can be established in the region. The forensic imaging resource sharing platform can include the facility access information, the training resources, the general guideline of forensic imaging performances, etc. Forensic imaging relies on instruments and technologies derived from the medical field. Any update or development of medical imaging technology may help to promote the development of imaging applications in forensics. With the maturity of deep learning technology [84,85] and artificial intelligence [86] technologies, they are promising for potential applications as a screening tool or in computer-aided diagnostics in forensic cases.
